# Mini-Implants Retaining Removable Partial Dentures in Subjects without Posterior Teeth: A 5-Year Prospective Study Comparing the Maxilla and the Mandible

**DOI:** 10.3390/medicina59020237

**Published:** 2023-01-27

**Authors:** Asja Celebic, Ines Kovacic, Nikola Petricevic, Dario Puljic, Aleksandra Popovac, Sanja Persic Kirsic

**Affiliations:** 1 Department of Removable Prosthodontics, School of Dental Medicine, University of Zagreb, 10000 Zagreb, Croatia; 2 School of Dental Medicine, University of Zagreb, 10000 Zagreb, Croatia; 3 Clinic for Dental Prosthetics, School of Dental Medicine, University of Belgrade, 11000 Belgrade, Serbia

**Keywords:** mini-implants, removable partial denture, Kennedy class I, clinical and radiological outcomes, peri implant marginal bone loss, modified plaque index, success, survival

## Abstract

*Background and objectives*: Long-term studies of clinical outcomes of mini-implants (MDIs) in the first premolar/canine sites retaining a bilateral free-ending removable partial dentures (RPD) in Kennedy class I subjects have not been well documented. The aim was to assess clinical outcomes in a prospective 5-year cohort study comparing the mandible and maxilla. *Material and Methods:* Participants (*n* = 92) who received two MDIs each and a new RPD were reviewed after one, three and five years. A total of 71 participants (82 mini-implants in the mandible; 58 in the maxilla) completed the study. Marginal bone level change, success, survival rates, Modified Plaque (MPI) and Bleeding Indices (MBI) were assessed. *Results:* The five-year success rate was 93.3% and 93.4% (*p* > 0.05), in the mandible and the maxilla, respectively. Mean peri-implant bone loss (MBL) increased significantly over five years (*p* < 0.01) to 0.50 mm in the mandible and 0.52 mm in the maxilla. Age had a significant effect on the MBL (higher rates in younger participants), while jaw of insertion, gender, and antagonistic jaw status did not. MPI and MBI were not significantly correlated with MBL. *Conclusions:* The insertion of two MDIs in previous first premolar/canine sites for retention of a free-end saddle RPD can be a successful treatment modality in subjects with narrow alveolar ridges.

## 1. Introduction

Treatment modalities in patients without posterior include multiple treatment options, such as the manufacture of conventional removable partial dentures (RPD) retained by clasps or precision attachments, implant-assisted removable partial dentures (IARPD), or implant-supported fixed partial dentures (IFPD) [1,2,3,4,5,6,7]. During masticatory loadings, free-end saddles of conventional RPDs move tissue-ward, thus enhancing the resorption of the underlying bone and, at the same time, compromising abutments through potentially destructive rotational forces [8,9]. A Kennedy class I situation can be transformed into a class III situation by the strategic addition of standard dimension dental implants, usually in the previous molar regions. This achieves a more favorable transmission of forces, reduces the movement of the direct retainer adjacent to the edentulous space, and decreases the stress magnitude in the periodontal ligament. The implant receives most of the load, reducing the amount of the displacement of free-end saddles [10]. Many studies revealed that tooth and implant-supported RPDs represent a reliable option with excellent prosthetic and implant survival rates and favorable rates for the abutments after five or more years of follow-up [2,10,11]. The IARPD also increases patient satisfaction due to improved comfort, support, and mastication [3,6,12].

In RPDs with distally placed implants (molar region), retentive clasps are often present on anterior abutments. However, implants can be placed more anteriorly, in an area adjacent to the abutment tooth to allow clasp omitting and the achievement of better aesthetic appearance, but still leaving the Kennedy class I situation. Recent studies revealed that the success of standard-size implants retaining IARPDs was associated with the strategic position. Strategic implant abutments adjacent to the natural tooth had similar or even higher success rates than those away from the natural tooth abutments [2,13,14].

Many long-term RPD wearers have insufficient bone volume for the insertion of standard dimension implants. Bone augmentation, a demanding and time-consuming procedure, is often rejected by patients. However, dental implant dimensions can be adapted, and implants of smaller diameter can be inserted into a narrow residual ridge without bone augmentation. Mini-implants (MDIs) are one-piece implants and belong to the first and the narrowest category (category 1) of narrow diameter implants (diameter ≤ 2.5 mm) [15]. The MDIs have been approved as a suitable treatment option for retention of mandibular complete overdentures in subjects with narrow ridges in many follow-up studies [16,17,18,19,20,21,22,23]. A recent study recommended MDIs even for the support of crowns or small bridges in the mandibular incisor region [24]. However, MDIs are associated with higher rates of marginal bone loss and significantly smaller success and survival rates in the maxilla when used for the retention of maxillary overdentures [25,26]. Reports about MDIs retaining RPDs are mostly limited to in vitro studies [27,28,29], case reports [30] or to small periods of observation [31,32,33,34]. One study reported patient satisfaction with MDIs, placed under existing RPDs, over the period of three years [35], and another reported on their stability and clinical outcomes [36]. Researchers mostly recommend longitudinal studies with a minimum of five years follow-up [37].

Therefore, the aim of this controlled prospective 5-year study was to assess clinical outcomes [marginal bone level (MBL) change, success and survival rates, oral hygiene maintenance, and prosthodontic maintenance] of mini-implants retaining a RPD in bilaterally edentulous patients without posterior teeth (Kennedy class I). The hypothesis was that the insertion of mini-implants in the first premolar or canine site may be a beneficial clinical option with predictable outcomes.

## 2. Materials and Methods

### 2.1. Study Design

The research was designed as a prospective clinical cohort study. It was performed between June 2015 and March 2022 with the approval from the Institution Ethics Committee (No. 05-PA-26-6/2015; School of Dental Medicine, University of Zagreb). The costs of the implants were covered by the research grant (Croatian Science Foundation, No. 1218/2014) and the costs of RPDs by the patient’s health insurance. After being fully informed about the treatment protocol and the risks and benefits, all participants provided informed consent. All participants were previous Kennedy class I clasp retained RPD wearers who were referred for new dentures. The determining factors for inclusion were: no posterior teeth present, the presence of three to six remaining teeth in the anterior region of the mandible or the maxilla, or of two canines only, and a presence of a narrow alveolar ridge (≤5.0 mm) at the sites of MDI placement, anatomical restrictions for implant placement in the posterior edentulous regions (e.g., alveolar nerve, the floor of the maxillary sinus), understanding treatment procedures, good attitude towards mini-implants, an independent living situation, and motor skills enabling oral hygiene maintenance [38]. Exclusion criteria were advanced periodontal disease of participants’ natural anterior dentition, flabby ridge or mucosa of the denture bearing area thicker than 4 mm, movable tissue attachments near the top of the ridge (when a participant refused surgical removal), a history of bruxism, a knife-edge shaped ridge at the place of planned implant insertion, or any disease that could affect wound healing or osseointegration. General inclusion/exclusion criteria were the same as those accepted for any implant placement [39]. Patients smoking up to 20 cigarettes per day and those with controlled diabetes melitus were not excluded.

**Figure 1:**
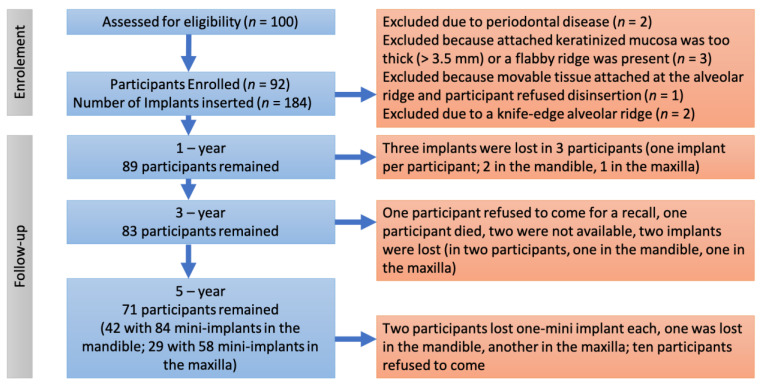
Flow chart of the study.

A study flow chart is presented in Figure 1. Alveolar length and width were assessed during clinical examination and were measured in panoramic radiographs and CBCTs. The mucosal thickness of a denture bearing area was assessed during clinical examination (by pressing a graduated probe toward the bone or by a sterile endodontic spreader with a rubber stop which punctured the overlying mucosa until the tip reached the bone).

### 2.2. Sample Size

A sample size calculation was based on the primary outcomes (peri-implant bone level changes, success, and survival rates of mini-implants) based on previously reported data for the mean MBL change in mandibular overdentures, which were retained by four MDIs [16,40,41,42,43].

Assuming there would be no difference between MDIs used for the retention of mandibular overdentures and MDIs used for retention of RPDs, the minimum sample size was 48 participants (24 for the mandible and 24 for the maxilla) with the accounted 30% dropout rate (alpha = 0.05, power = 80%). It was decided to include twice as many participants in order to account for the duration of the follow-up.

### 2.3. Surgical Protocol

Implant placements were performed by two residents of oral surgery under the supervision of two experienced specialists in oral surgery and prosthodontics. The MDI dimensions (10, 12, or 14 mm long and 2.0 or 2.5 mm wide; Dentium, Seoul, South Korea) were chosen after measurement of the available bone on CBCT scans. The flapless surgery was performed using the calibrated burs, a physiodispenser (W&H Implantmed, GmbH, Austria), and a saline solution for external drill cooling under local anesthesia (4% articaine or 3% mepivapacaine, 3M, Germany). The bone was prepared to a depth of one-half or two-thirds of implant length depending on the bone density (D3 or D2). The diameter of the drill for bone preparation was smaller than the width of the implant by 0.3–0.7 mm. The self-tapping insertion technique was used, first by a thumb wrench and finally the torque wrench. One MDI was inserted on each side of the edentulous ridge (two MDIs per a patient) in previous sites of the first premolars or canines, one or two tooth lengths distally from the patient’s last remaining tooth. The MDIs were placed parallel to each other and parallel to the planned path of a new RPD insertion. In cases when the insertion torque was ≥35 Ncm, the implants were loaded early (at 6–8 weeks), and in cases when the insertion torque was lower than 35 Ncm, the MDIs were loaded three months after insertion. The standard postsurgical instructions with a detailed written description of how to maintain oral hygiene were given to the participants.

### 2.4. Prosthodontic Protocol

**Figure 2:**
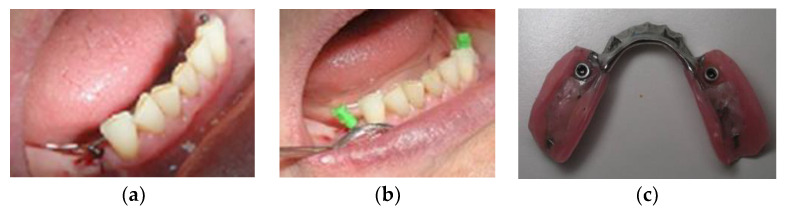
(**a**) Mini-implants after insertion; (**b**) Transfer caps attached to mini-implants before custom impression; (**c**) Denture at delivery with metal-housing and “o”-rings.

The residents in Prosthodontics made all RPDs under the supervision of two experienced specialists. The Co-Cr framework was made to reinforce RPDs to prevent denture fractures. The mandibular RPDs had lingual plate (linguoplate) major connectors with cingulum rests on the remaining anterior teeth. The maxillary RPDs had either a full palatal coverage or a U-shaped major connector with cingulum rests. Individual impressions in custom trays were obtained with transfer caps attached to the MDIs (Figure 2). Laboratory analogs were inserted into transfer caps, and casts were poured in hard stone. Matrices (metal housing with “o”-rings) were attached to the MDI analogs. After the metal framework manufacture and denture teeth set-up, the RPDs were processed. During processing, “o”-rings had to be removed from the metal housing, but were returned afterwards. Upon delivery, the new RPDs were adjusted over 15 days.

### 2.5. Clinical and Radiographic Evaluation

**Figure 3:**
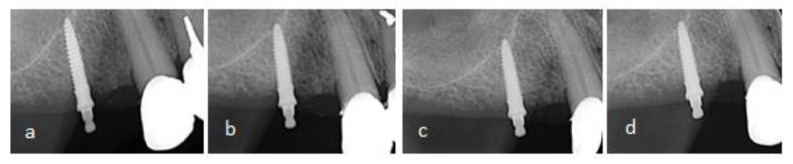
Marginal bone level (MBL) at mesial and distal sites of a MDI, (**a**) after insertion, (**b**) after one year, (**c**) after three years, (**d**) after five years of function.

After MDI insertion, immediate follow-up radiographs were done to validate the correct implant position. The long-cone paralleling technique (Minray Soredex Intraoral, Tuusula, Finland, 70 kV, 0.16 mAs) was used to obtain digital intraoral radiographs. For reproducible projections, a film holder (X-ray holder, Super-Bite^®^, Kerr USA, Orange, CA, USA) with a customized silicone index for each participant was used. The marginal bone level (MBL) was measured using Scanora software (v. 5.1, Soredex, Tuusula, Finland) at 10× zoom-in at the mesial and distal sites of each MDI rounding up the values to the nearest 0.1 mm. The mesial and distal bone height measurements were averaged to obtain the mean MBL change. The magnification error was corrected using the formula reported by Yoo RH et al. [44]. The bone level at an RPD delivery was considered as the baseline. When any part of the smooth MDI surface was slightly submerged during surgery, bone loss until it reached the roughened threaded surface was considered bone remodeling. The bone loss was measured apically from the roughened threaded surface. In cases when any of the roughened threads were not in the bone after surgery, the first bone-to-implant contact was considered as the baseline. Successive intraoral radiographs are presented in Figure 3.

Modified Plaque Index (MPI) and Modified Bleeding Index (MBI), according to Mombelli (scores 0–3) were also assessed [45]. Any problems with MDIs during the follow-up period (pain, exudate, mobility, fracture, loss) or with retention elements (loss or “o”-ring changes, metal housing loosening) were recorded. Implant success and survival rates were assessed at the 1-year, 3-year, and 5-year follow-up examinations. The assessments were based on the Consensus Conference of the International Congress of Oral Implantology in Pisa, Italy in 2007 [46]. Implants were considered successful when participants had no ongoing pain or history of pain, no foreign body sensation or dysesthesia, no recurrent peri-implant infections, no implant mobility, or continuous radiolucency > 2 mm, and when the implant would be suitable for a prosthodontic restoration. Two different survival categories were satisfactory survival or compromised survival. Successful survival was described as a peri-implant marginal bone loss slightly > 2 mm either at the mesial or distal site, but not requiring any clinical management. An implant having less than ideal conditions and requiring serious clinical treatment to reduce the risk of failure was considered to have a compromised survival. Implant failure was specified when an implant required removal or had already been lost.

### 2.6. Prosthodontic Maintenance

Complications and repairs regarding partial removable dentures and attachments were recorded during the study (e.g., loosening of metal housing, loss of “o” rings or a need for replacement due to wear, fracture of a denture, need for relining, detachment of artificial teeth).

### 2.7. Statistical Analysis

A statistical analysis was performed using the statistical software (SPSS for Windows, version 20). *X*^2^ tests were applied to test the significance of the difference in the number of participants between the groups (mandible vs. maxilla). The MBL changes at different timepoints (one, three, and five years) were analyzed (α = 0.05) for normality (one sample Kolmogorov-Smirnov test) and homogeneity (Levine). The correlation between marginal bone level change and the participant’s age was calculated. A repeated-measures analysis was performed for the MBL changes at the three timepoints (within-subject effect) and accounting for three factors: jaw of implant insertion, gender, and antagonistic jaw status (between-subject effect) with the age as a covariate. Based on the interpretation of *p* values from the Mauchly analyses, the Greenhouse-Geisser correction was applied when sphericity was not assumed (*p* < 0.05). When significant effects for the variables or their interaction was observed, the Bonferroni post-hoc tests were applied to point-out significant effects (*p* < 0.05). Kaplan–Meier curves were made for survival and success reports (mini-implant failures/compromised survivals were accounted as one category, while successful implants/successful survivors were accounted as another category). A comparison of failure/compromised survival and success/satisfactory survived mini-implants between the mandible and the maxilla was made using the log-rank Mantel-Cox test. Friedman’s non-parametric test for related samples was performed to test the significance of the differences for the MPI and the MBI over time.

## 3. Results

From 92 participants included at baseline (each participant received two MDIs and a new RPD; 53 in the mandible, 39 in the maxilla), 71 of them completed a full 5-year observation period [42 participants with mini-implants in the mandible (84 MDIs), and 29 (58 MDIs) with mini-implants in the maxilla]. There were no significant age (t [69] = 1.51), gender (*X*^2^ = 0.19), or antagonistic jaw status (*X*^2^ = 5.48) differences between participants who received implants in the mandible or maxilla (*p* > 0.05) (Table 1). Descriptive statistics for MBL changes at three different follow-up timepoints can be found in Table 2, grouped by insertion site, gender, antagonistic jaw status (complete denture, removable partial denture, natural teeth or fixed partial denture). Peri-implant marginal bone loss increased over the observation period. It increased both in the mandible and maxilla, in female and male participants, and in participants with different antagonistic jaw status. Participants’ age had a significantly negative correlation with peri-implant marginal bone loss at each timepoint (1-year: r = −0.357, *p* = 0.002; 3-year: r = −0.352, *p* = 0.003; 5-year: r = −0.354, *p* = 0.002). The repeated-measures analysis (Within-Subject Effects) revealed that peri-implant marginal bone loss increased significantly over time [Greenhouse-Geisser; F (df 1.51) = 6.54; *p* = 0.01, Ƞ^2^ = 0.097] with significant differences between the 1-year and 3-year, 1-year and 5-year, and 3-year and 5-year follow-ups. The MBL change*Age also showed a significant effect [Greenhouse-Geisser F (df 1.51) = 4.41, *p* = 0.034, Ƞ^2^ = 0.097]. Other factors (MBL*jaw of insertion; MBL*gender, MBL*antagonistic jaw status) had no significant effects (*p* > 0.05). Tests of between-subjects effects revealed that only age (covariate) (F = 9.17, *p* = 0.004, Ƞ^2^ = 1.31) had a significant effect, with higher rates of marginal peri-implant bone loss in younger participants. Jaw of insertion (*p* = 0.54), gender (*p* = 0.96), and antagonistic jaw status (*p* = 0.77) showed no significant effects.

**Figure 4:**
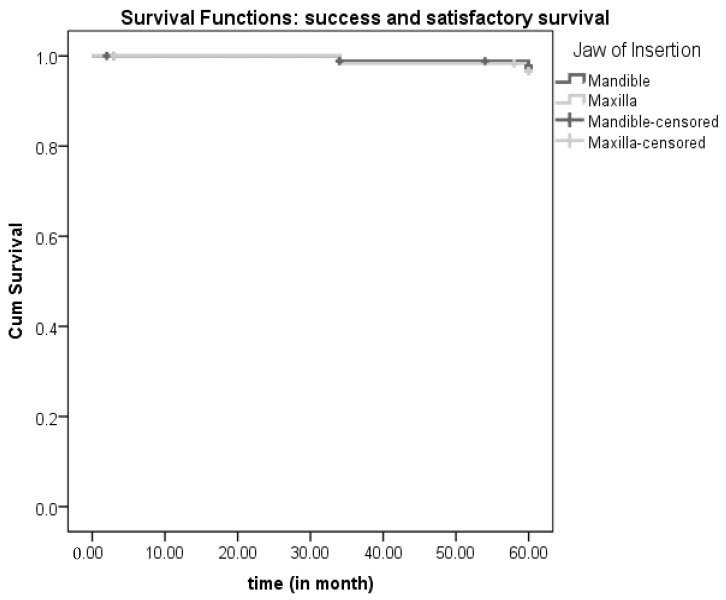
Kaplan Meier curves for success/satisfactory survival and failure/compromised survival over 5-years of follow-up.

Three implants were lost during the first year in three different participants (two in the mandible, one in the maxilla) within the first four months after insertion, soon after loading. All other patients (*n* = 89) were available at the 1-year follow-up examination. They all had successful implants, except for the two implants in two different patients listed in the satisfactory survival category (one in the maxilla and another in the mandible). At the 3-year follow-up examination, four patients were lost to follow-up (one died, two refused to come due to general health problems, and one was not available). Furthermore, two implants were categorized as compromised survivals in two different patients (one in the maxilla, one in the mandible). One patient had a failed implant in the mandible and one implant that belonged to the compromised survival group. A total of 83 participants remained with successful implants (or satisfactory survivals). A total of 10 patients did not respond at the 5-year examination (due to fear of COVID-19 infection). Two mini-implants were lost in two patients (one in the maxilla, one in the mandible (fractured), and two implants in two patients were grouped in the compromised survival category (one in the maxilla, one in the mandible). A total of 71 patients with 142 implants successfully reached the final follow-up at 5 years. A total of 140 implants in 70 participants were successful or satisfactory survivals, while two implants were described as compromised survivals. Rates of success/satisfactory survival in the mandible and maxilla can be found in Table 3. Kaplan-Meier curves are shown in Figure 4. The Log-Rank (Mantel-Cox) test revealed no significant difference between the mandible and maxilla when comparing the number of events (failure/compromised survival rates vs. success/satisfactory survival) at the implant level (*X*^2^ = 0.003; df = 1; *p* = 0.954, N.S.). Moreover, there was no significant difference between the groups (mandible vs. maxilla) either when analyzing only the event failure *X*^2^ = 0.144; df = 1; *p* = 0.705, N.S.) or only the event compromised survival (*X*^2^ = 0.014; df = 1; *p* = 0.708, N.S.).

The frequency of modified plaque and bleeding indices at each of the follow-up examinations are presented in Table 4. Oral hygiene worsened and both MPI (*X*^2^ = 6.33; *p* = 0.042) and MBI (*X*^2^ = 13.07; *p* = 0.001) increased significantly over the observation period. The 5-year modified plaque index (Spearman’s rho = −0.42; *p* = 0.730), and the 5-year modified bleeding index (Spearman’s rho = −0.02; *p* = 0.885) were negatively, although not significantly (*p* > 0.05), correlated with marginal bone loss after five years.

Considering prosthodontic maintenance, it is important to mention that no RPD fractures were registered. One denture was relined after one year, three dentures were relined after three years, and seven RPDs were relined after five years in function. Two metal housings were loosened and had to be re-attached using a self-curing resin in the third year. Only one “o”-ring needed replacement in the first year (it was lost), while 62 “o”-ring replacements were done at the 3-year examination (three “o”-rings were lost). During the 5-year follow-up examination it was decided to replace all “o”-rings which had not been replaced previously. Two artificial teeth were detached from the dentures and were repaired, one in the third year and another in the fourth year. Two natural teeth in two patients were lost, one in the fourth year, and another in the fifth year, due to cervical caries (lost teeth were not adjacent to MDIs), and were replaced in the respective dentures.

## 4. Discussion

This prospective cohort study evaluated a new approach in the treatment of Kennedy class I patients with narrow ridges without posterior teeth by means of two mini-implants not wider than 2.5 mm inserted in the previous sites of the first premolars or canines for retention of free-end saddle RPDs. The results revealed small rates of peri-implant bone loss, not different between the maxilla and the mandible, and acceptable 5-year success rates (93.3% in the mandible, 93.4% in the maxilla).

The benefits of IFPDs in Kennedy Class I patients have been extensively described in the dental literature [7,47]. However, many patients are not candidates for IFPD treatment due to restrictions of their posterior alveolar ridge anatomy, financial status, fear of demanding surgical interventions, or co-morbidities that restrict demanding surgical procedures and healing [48,49,50]. Therefore, the manufacture of conventional RPD can be an alternative. However, in conventional RPD wearers (Kennedy class I patients with linear support), saddles move tissue-wards promoting bone atrophy and cause damage to the periodontal tissue of abutment teeth due to rotational forces [8,9]. The visibility of anterior abutment clasps causes additional aesthetic concerns [1]. The insertion of standard dimension implants in strategic positions under a removable partial denture significantly improves dental-patient reported outcome measures and masticatory performance [2,9,10,11,12,14], but many patients have anatomical restrictions (narrow ridges) for standard-sized implant insertion. Therefore, the insertion of mini-implants can be a beneficial solution. It is also a cost-effective method [51] that is frequently performed by a flapless surgery which provokes less pain than the open-flap insertion of standard-sized implants [48,49], favoring the MDIs for geriatric patients whose bone volume is usually reduced. However, due to a lack of clinical outcomes in longitudinal clinical studies on MDIs retaining an RPD, the longitudinal prospective cohort study was designed, comparing the mandible and maxilla. Participants whose alveolar ridge was wider than 5.0 mm were excluded because they were able to receive implants of standard dimensions. All participants had narrow ridges and were previous conventional clasp retained RPD wearers. They received small diameter MDIs (Dentium, South Korea, 2.0 or 2.5 mm wide; 10-14 mm long), to maintain at least 1 mm of crestal bone around the implant [52]. The mini-implants were inserted in the sites of previous first premolars or canines, anteriorly from the mental foramen or below and anteriorly of the sinus floor, thus leaving the Kennedy type I situation. As the MDIs were aimed at retention, a denture was manufactured without clasps. However, by MDI insertion, linear support was converted into more favorable quadrangular support, providing better force distribution and resistance to displacement by functional stresses. The retention system was a metal housing with a rubber “o”-ring, allowing distal-extension saddles a certain amount of tissue-ward movement without loading MDIs. Although the benefits of standard-sized dental implants inserted in previous molar sites are well-known [53,54], some papers described their placement in previous first premolar sites and found out that they led to better relief in the distal abutment tooth [2,14,55,56]. The results of this study, with category 1 narrow implants (MDIs) in the sites of the first premolars or canines, are in agreement with those studies, as well as with a recent in vitro study utilizing mini-implants [27]. That study revealed that mesially (first premolar site) placed mini-implants showed the lowest strain around abutment teeth and concluded that under a favorable biting force, mini-implants were an option to assist distal extension removable partial dentures. In the present study, during a 5-year follow-up, none of the teeth adjacent to an MDI were lost. Only two teeth were lost, and they were distant from the mini-implants due to caries in the 4th and the 5th year. 

The metal housing with “o”-rings belongs to a resilient attachment system [57] allowing some bending movements to a free-end saddle without overloading implants or abutment teeth. Participants with resilient or too thick mucosa (that would allow more tissue-ward RPD settling) were excluded from this clinical trial, as were bruxers. The mini-implant success and survival rates, obtained in the present 5-year study, were similar or even better than those reported by Threeburuth et al. [33] in their 1-year study. However, they placed 3 mm wide implants in previous first molar sites with an equator attachment system (stud attachment), while more resilient attachments were used in this study at the first premolar sites. Implant inclination is also an essential factor for the distribution of stress and tension around implants, as tensions are higher in inclined implants [58]. Therefore, special attention was paid during the insertion of two MDIs to be parallel to each other and parallel to the path of an RPD insertion. The metal housing with “o”-rings also accommodated up to ±15 degrees of tilting angles between MDIs and a pathway of an RPD insertion. The attachment height can also have an influence on the bending moment [59]. It has been described that high-profile attachments cause higher bending movements than low-profile attachments [59]. Low profile and resilient attachments were selected in this study. In the worst-case scenario, only 6 mm of polished MDI surface was above the bone level, allowing a good ratio between the part above the bone and the part of implant in the bone.

The lower amount of peri-implant bone loss in this study, compared to peri-implant bone loss in complete mandibular overdentures during the same period of observation, can be attributed to the better stability of RPDs due to the presence of anterior teeth and a linguoplate major connector with cingulum rests, which additionally stabilized the RPD [41,58,60,61,62,63,64]. Although studies on mini-implants retaining maxillary complete overdentures [25,26,65] reported lower success rates and considerably higher amounts of peri-implant bone loss when compared to mandibular overdentures, this was not found in the present study. This may be attributed to the fact that anterior maxillary dentition offers good RPD stability by supporting cingulum rests, to the exclusion of participants with too resilient and too thick mucosa, to the exclusion of those with bruxing habits, and to the utilization of resilient attachments. Additionally, no D4 bone density existed in the first premolar/canine sites in the maxilla. Mundt et al. [36] reported the 3-year survival rates of strategic mini-implants with immediate or delayed loading under removable partial dentures to be better in the mandible (99%, one failure) than in the maxilla (87%, eleven failures). In this study, only early or delayed loading was performed, and our exclusion criteria omitted bruxers and patients with too thick or flabby mucosa, which could elicit MDI overloading throughout extensive RPD movements.

At the 1-year follow-up observation all participants responded, but three of them were excluded, as they lost one MDI each soon after loading. Four patients were lost to follow-up by year three, and ten patients by year five. It is likely that the motivation for follow-up reduced over time, bearing in mind that most of the participants were over 65 years of age with co-morbidities and fell within the high-risk patient category during the COVID-19 pandemic, when the follow-up took place. None of the MDIs were lost during surgery; however, three mini-implants were lost very soon after loading, probably due to their improper position in the bone bed. This may be attributed to flapless surgery or to the insufficient experience of the residents in oral surgery who placed MDIs, and can be considered as a surgical complication. Open-flap surgery would probably result in a higher success rate after loading [66]. Lost MDIs (or fractured) after three years could be ascribed to the increased movements of an RPD due to bone atrophy (RPD not relined in time) or to not changing resilient “o”-rings in time, thus overloading mini-implants.

Regarding oral hygiene maintenance (MPI, MBI), significantly more plaque and bleeding on probing was found later in the follow-up, likely due to participants’ reduced motivation over time. MPI and MBI were not significantly correlated with the amount of peri-implant bone loss after five years, highlighting that other reasons besides oral hygiene (e.g., distal-extension free-end saddle settling and/or MDI overloads) may be more important factors for peri-implant bone loss.

The fact that there was no significant gender difference in the amount of peri-implant bone loss is in line with other studies [67,68]. Younger patients showed a significantly more pronounced amount of peri-implant bone loss, which is in line with a study conducted by Jemt [69]. Younger patients probably develop higher occlusal forces because of chewing more vigorously, and there is a lack of proprioception of the implants [70]. However, the magnitude of occlusal forces was not measured, which is a limitation of this study, although different antagonistic jaw status (FPD, RPC, CD) did not elicit significant effects on peri-implant bone [71].

No fractures of denture bases were registered during the observation period thanks to reinforcement of a denture base by a metal framework. Most of the “o”-ring replacements were done due to wear and calculus formation; only several “o”-rings fell out of metal housings. In spite of the fact that the manufacturer recommends the changing of the “o”-rings once a year, all “o”-rings were only changed after five years. It is important to change “o”-rings and/or reline a denture to prevent MDI’s overloads and unwanted forces. More effort should be invested to motivate the patients to come for regular follow-ups and understand the importance of an RPD relining and “o”-ring replacement.

Limitations of the study, besides possible variations in the amount of chewing forces, may include the possible insufficient experience of the residents who inserted MDIs (although they were supervised by experienced specialists), the 2-dimensional assessment of peri-implant bone loss, small variations in dimensions of MDIs, and small variations of the thickness of keratinized mucosa around implants [71]. The existence of chronic stress and depression, which could influence peri-implant disease and bone loss through different mechanisms [72] was not evaluated. The strength of the study is the 5-year observation period and the controlled and prospective study design. 

This 5-year study generally revealed very good intermediate-term clinical results. Low levels of peri-implant bone loss (−0.50 mm in the mandible; 0.52 mm in the maxilla), and acceptable success rates with no significant difference between the jaws over the 5-year period validated this treatment possibility. Based on the results of the present study, and keeping in mind that it is less expensive to insert two MDIs when only some anterior teeth remain in the jaw than it is to prepare them for an FPD and include precision attachments, and that some slightly movable teeth can even be left and easily replaced in the existing RPD, there is a rationale to use mini-implants in the Kennedy Class I patients for a RPD retention. 

## 5. Conclusions

Within the limitations of this 5-year follow-up clinical prospective study, mini-implants in the previous sites of first premolars or canines inserted for the retention of RPDs in Kennedy class I patients provided very good clinical outcomes due to the small rate of marginal bone loss and acceptable survival and success rates. This treatment modality can represent a low cost and minimally invasive solution. However, further studies, and those covering a longer observation period, may be required to confirm the results of this study.

## Figures and Tables

**Table 1:** Summary of the study variables and the significance the difference between the groups of the participants who completed a 5-year follow-up.

Variable	All Participants	Mandible	Maxilla	*p*-Value
Sample size (n)	71 (100%)	42 (59.2%)	29 (40.8%)	
Gender, female	57 (80.3)	33 (57.9%)	24 (42.1%)	0.770 N.S.
Age, years	67.55 ± 7.93	66.38 ± 8.61	69.24 ± 6.61	0.140 N.S.
Age group				0.131 NS
<60 years	12 (16.9%)	10 (83.3%)	2 (16.7%)	
61–70 years	34 (47.9%)	17 (50.0%)	17 (50.0%)	
>70 years	25 (35.2%)	15 (60.0%)	10 (40.0%)	
Antagonistic jaw				0.061 N.S.
Complete denture	26 (36.6%)	15 (57.7%)	11 (42.3%)	
RPD	31 (43.7%)	15 (48.4%)	16 (51.6%)	
Natural teeth/FPD	14 (19.7)	12 (85.7%)	2 (14.3%)	
Number of mini dental implant at baseline	142	84	58	
Number of mini dental implants lost to follow-up	5	3	2	

RPD = removable partial denture, FPD = fixed partial denture; N.S. = not significant (*p* > 0.05).

**Table 2:** Descriptive statistics for marginal bone level (MBL) changes over the observation period.

		MBL Change	N	x	SD	95% Confidence Interval
**All participants**		1 year	71	−0.22	0.36	0.13–0.30
		3 years	71	−0.38	0.56	0.25–0.52
		5 years	71	−0.51	0.66	0.35–0.67
**Jaw** **of** **Insertion**	Mandible	1 year	42	−0.22	0.32	0.10–0.33
		3 years	42	−0.37	0.48	0.19–0.54
		5 years	42	−0.50	0.56	0.29–0.70
	Maxilla	1 year	29	−0.22	0.41	0.08–0.35
		3 years	29	−0.40	0.67	0.19–0.61
		5 years	29	−0.52	0.78	0.28–0.77
**Gender**	Female	1 year	57	−0.24	0.37	0.13–0.36
		3 years	57	−0.42	0.59	0.19–0.54
		5 years	57	−0.54	0.67	0.27–0.68
	Male	1 year	14	−0.22	0.26	0.03–0.41
		3 years	14	−0.32	0.41	0.06–0.65
		5 years	14	−0.51	0.57	0.17–0.86
**Antagonistic** **jaw** **status**	Complete denture	1 year	26	−0.16	0.26	0.06–0.26
		3 years	26	−0.25	0.34	0.11–0.38
		5 years	26	−0.39	0.47	0.20–0.58
	Removable partial denture	1 year	31	−0.24	0.40	0.09–0.38
		3 years	31	−0.51	0.70	0.25–0.77
		5 years	31	−0.64	0.80	0.34–0.93
	Natural teeth or Fixed partial denture	1 year	14	−0.28	0.43	0.03–0.52
		3 years	14	−0.35	0.53	0.04–0.65
		5 years	14	−0.44	0.59	0.09–0.78

N = number of cases; x = mean value; SD = standard deviation.

**Table 3:** Survival analysis of mini-implants after five years in function (failure, compromised survival, satisfactory survival, and success) at the implant level.

			Successful Implants		95% Confidence Interval (Time in Month)	
Group	Number of implants (baseline)	Number of Events	n	Rate (%)	Lower bound	Upper bound
Mandible	106	6 (6.7%)	83	93.3	55.94	60.17
	Failure		Success or satisfactory survival			
Maxilla	78	4 (6.6%)	57	93.4	56.34	60.90
	Failure or compromised survival		Success or satisfactory survival			
Overall	184	10 (6.7%)	140	93.3	56.80	59.76
	Failure or compromised survival		Success or satisfactory survival			

14 participants with 28 mini-implants were lost to follow-up.

**Table 4:** Frequencies of modified plaque and bleeding indices over time.

P L A Q U E I N D E X	Modified Plaque Index: 1-year follow-up	Degree	Frequency (n)	Percent (%)
		0	21	29.6
		1	30	42.3
		2	20	28.2
	Modified Plaque Index: 3-year follow-up	0	20	28.2
		1	31	43.7
		2	15	21.1
		3	5	7.0
	Modified Plaque Index: 5-year follow-up	0	16	22.5
		1	33	46.5
		2	17	23.9
		3	4	5.6
B L E E D I N G I N D E X	Modified Bleeding Index: 1-year follow-up	Degree	Frequency (n)	Percent (%)
		0	27	38.0
		1	35	49.3
		2	9	12.7
	Modified Bleeding Index: 3-year follow-up	0	19	26.8
		1	35	49.3
		2	17	23.9
	Modified Bleeding Index: 5-year follow-up *	0	16	22.5
		1	36	50.7
		2	18	25.4

*—one case missing (1.4%).

## Data Availability

Data available on request due to privacy restrictions.
